# High Malignancy Risk and Its Predictors in South Indian Patients With Bethesda II Thyroid Nodules

**DOI:** 10.7759/cureus.54923

**Published:** 2024-02-26

**Authors:** Sunanda Tirupati, Pradeep Puthenveetil, Shilpa Lakkundi, Anudeep Gaddam, Vijaya Sarathi

**Affiliations:** 1 Endocrinology, Narayana Medical College, Nellore, IND; 2 Endocrine Surgery, Baby Memorial Hospital, Kozhikode, IND; 3 Pathology, Vydehi Institute of Medical Sciences and Research Centre, Bangalore, IND; 4 Endocrinology and Diabetes, Kasturba Medical College, Manipal, IND; 5 Endocrinology and Diabetes, Narayana Medical College, Nellore, IND; 6 Endocrinology, Vydehi Institute of Medical Sciences and Research Centre, Bangalore, IND

**Keywords:** papillary carcinoma of thyroid, southern india, malignancy risk, thyroid nodule size, thyroid cancer

## Abstract

Background: Global data reports a low malignancy risk, whereas regional data report a variable risk of malignancy in Bethesda II thyroid nodules. The limited availability of surgical histopathology might have underestimated the risk of malignancy. Here, we report the prevalence of malignancy and its predictors in Bethesda II thyroid nodules for which the surgical histopathological diagnosis was available.

Methods: This retrospective study was done at a tertiary healthcare center in South India between January 2008 and September 2015. Case records of adults with thyroid nodules who underwent surgery were collected. Patients with inadequate data were excluded from the study. The data was analyzed using SPSS version 21.0 and a p-value of < 0.05 was considered statistically significant.

Results: A total of 563 patients were included in the study with a mean age of 36±12 years. Serum thyrotropin (TSH) was low in 87 (15.4%) patients whereas 362 (64.2%) patients had multinodular goiter (MNG). Sonographic evidence of suspicious cervical lymph node and microcalcification was seen in four (0.7%) and 48 (8.5%) patients, respectively. A total of 48 (8.5%) patients had thyroid carcinoma in the final histopathology. Of these, 42 (87.5%) had papillary thyroid carcinoma, five (10.4%) had follicular thyroid carcinoma and one (4.1%) had anaplastic carcinoma. Age, gender, and maximum nodule size were not associated with malignancy. Thyrotoxicosis was negatively associated with malignancy whereas multi-nodularity, thyroid calcification, or suspicious cervical lymph node on ultrasound and total thyroidectomy were positively associated with malignancy on univariate analysis. On binary logistic regression, only the former four, but not total thyroidectomy, were independent predictors of malignancy.

Conclusions: We report a high (8.5%) prevalence of malignancy among South Indian patients with Bethesda II thyroid nodules. Thyroid microcalcification, presence of suspicious cervical lymph node on ultrasound, and multinodularity were associated with high and suppressed TSH with low risk of malignancy. Further prospective studies are warranted to confirm the study observations.

## Introduction

Globally, in 2020, the age-standardized incidence rates of thyroid cancer were 10.1 per 100,000 women and 3.1 per 100,000 men [1]. The incidence of thyroid cancer increased in the United States by 3.6% per year between 1974 and 2013, which was coupled with an increased thyroid cancer mortality rate for advanced-stage papillary thyroid cancer (PTC) [2]. An analysis reported a similar trend in India, with an increase in the age-adjusted incidence rate of thyroid cancer of 37% in women and 27% in men between 2006 and 2008, and 2012 and 2014 [3]. In a recent report from the Indian National Cancer Registry, thyroid cancer was the sixth most common cancer in females overall (3.6%) and the second most common (12.2%) cancer in young females (15-39 years of age) [4].

A recent meta-analysis reported thyroid nodules in 24.83% (95% CI 21.44-28.55) of the general population, regardless of the diagnostic techniques, with a higher prevalence in women than men (36.51% vs. 23.47%, p <0.01). A trend for an increase in the prevalence of thyroid nodules was also noted between 2000 and 2011, and 2012 and 2022 (21.53% vs. 29.29%, p = 0.02) [5]. The data on the prevalence of thyroid nodules in India are limited. A study from Kerala reported a prevalence of clinically detectable goiter of 12.2% [6]. Another recent study from Kerala found a sonological prevalence of thyroid nodules ≥ 1 cm in ~14% of the adult population [7]. Recognition of thyroid nodules is associated with a concern for malignancy, which occurs in 7-15% of nodules [8]. The most important task in patients with thyroid nodules is to differentiate those who have a low risk of malignancy from those who have a low risk.

The assessment of cancer risk in a thyroid nodule permits a cost-effective approach to selecting patients for surgery or observation. The initial evaluation measurement of serum thyrotropin (TSH) and ultrasonogram were used to obtain the Thyroid Imaging Reporting and Data System (TIRADS) and size for each nodule [8]. These data guide the selection of nodules for further fine needle aspiration cytology (FNAC). Based on FNAC findings, the nodules are categorized into six categories with different malignancy risks by The Bethesda System for Reporting Thyroid Cytology (TBSRTC) guidelines [9]. The sensitivity and specificity of palpation-guided thyroid fine needle aspiration (FNA) are 76% (95% CI, 49-89%) and 77% (95% CI, 56-95%), respectively, whereas those for ultrasound-guided FNA are 90% (95% CI, 81-95%) and 80% (95% CI, 66-89%), respectively, to assess the risk of malignancy [10].

The TBSRTC guidelines estimate a low malignancy risk (0-3%) in Bethesda II nodules [9]. Studies from India report variable risk of malignancy (0-13%) in Bethesda II thyroid nodules [11]. The inference from the majority of these studies is limited by the availability of surgical histopathology in only a proportion of these patients, which might underestimate the risk of malignancy [11]. Here, we report the prevalence of malignancy and its predictors in Bethesda II thyroid nodules for which the surgical histopathological diagnosis was available.

This article was previously presented as a meeting abstract at the ITSCON on May 18, 2019.

## Materials and methods

This retrospective study was performed at a tertiary healthcare center in South India. Case records of adults with thyroid nodules who underwent surgery between January 2008 and September 2015 and had a preoperative FNAC report of Bethesda II were reviewed. Demographic details (age, sex), family history of thyroid malignancy, size of the largest nodule on ultrasound, lymph node involvement, and evidence of calcification or neck nodes on ultrasound were recorded. Patients with missing above-mentioned data were excluded from the study. The institutional ethics committee of Narayana Medical College and Hospital (NMC/IEC/2019/11 meeting held on 15-11-2019) approved the study. A waiver for consent was granted given the retrospective nature of the study.

The Departments of Endocrine Surgery and Endocrinology referred all patients to the Department of Radiology for ultrasound-guided FNAC of thyroid nodules, where samples were collected and transferred to the Department of Pathology. All slides were reviewed by a cytopathologist. Subsequently, patients underwent surgery at the discretion of the operating endocrine surgeon. Surgical specimens were sent to the Department of Pathology for the final histopathological diagnosis.

The data were entered into Microsoft Excel and analyzed using Statistical Package for the Social Sciences (SPSS) version 21.0. Continuous data are expressed as the mean±SD, whereas categorical variables are expressed as absolute numbers and percentages. The continuous variables between the two groups were compared using independent t-tests, whereas the categorical variables were compared using chi-square tests. Binary logistic regression was used to calculate the odds ratio. A p-value of < 0.05 was considered statistically significant.

## Results

A total of 563 patients (49 males) were included in the study (Table 1), with a mean age of 36± 12 years. Serum TSH was low in 87 (15.4%) patients, whereas 362 (64.2%) patients had multinodular goiter (MNG). The mean size of the largest nodule in the overall study population was 3.65±1.89 cm. Sonographic evidence of suspicious cervical lymph nodes and microcalcifications was observed in four (0.7%) and 48 (8.5%) patients, respectively. Total thyroidectomy was conducted in 346 (61.4%) patients, including four with additional cervical lymph node clearance, whereas others underwent hemithyroidectomy.

**Figure 1:**
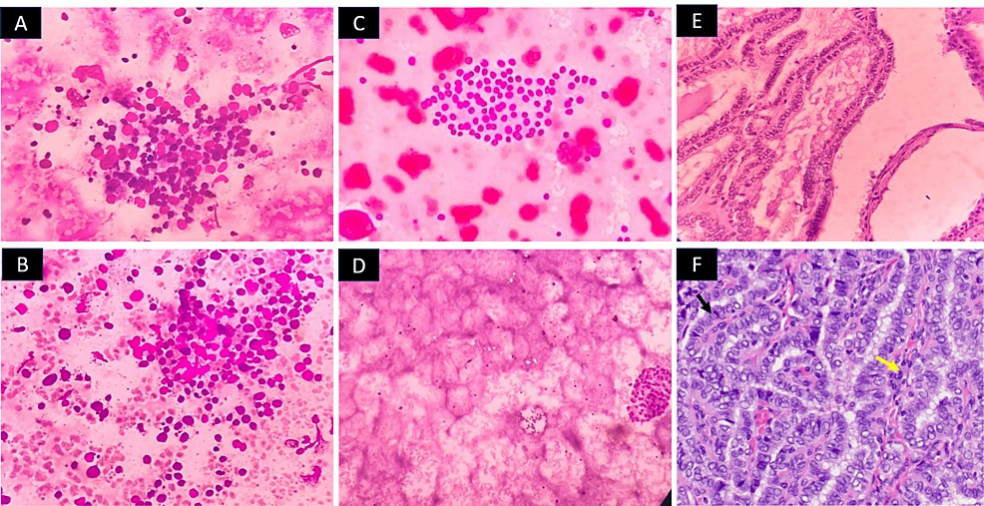
Fine aspiration cytology images of three representative patients with thyroid nodules. Panels A and B: Lymphocytic thyroiditis (The Bethesda System for Reporting Thyroid Cytology (TBSRTC) II) in representative case 1—(A) Lymphocytes infiltrating follicular epithelial cells (H&E, 10X), and (B) Lymphoid cells of varying stages of maturation infiltrating follicular epithelial cells (H&E, 10X); Panels C and D: Colloid goiter (TBSRTC II) in representative case 2—(C) Flat sheets with evenly spaced follicular cells, with pigment-laden macrophages (H&E,10X), and (D) A follicular cell cluster with a watery thin colloid background (H&E, 10X); Panels E and F: Papillary thyroid carcinoma (TBSRTC IV) in representative case 3—(E) True papillary fronds (H&E, 10X) with (F) pronounced nuclear features including nuclear grooving (black arrow), overlapping and clearing (yellow arrow) (H&E, 40X).

A total of 48 (8.5%) patients had thyroid carcinoma in the final histopathology. Of these, 42 (87.5%) had PTC, five (10.4%) had follicular thyroid carcinoma, and one (4.1%) had anaplastic carcinoma. Representative images of FNAC of the thyroid in a few representative cases are shown in Figure 1. 

Multinodularity, thyroid calcification, and suspicious cervical lymph nodes on the ultrasound and total thyroidectomy were positively associated with malignancy, whereas thyrotoxicosis was negatively associated with malignancy. Age, sex, and maximum nodule size were not associated with malignancy (Table 1). In binary logistic regression analysis (Table 2), multinodularity, thyroid calcification, or suspicious cervical lymph nodes on ultrasound were positive predictors, while suppressed TSH was a negative predictor of malignancy.

**Table 1:** Demographic, laboratory, sonological, and management characteristics of the study population USG: Ultrasonogram p-value of < 0.05 is considered significant.

Parameters	Total (n=563)	Benign (n=515)	Malignancy (n=48)	p-value
Male, n (%)	49 (8.7)	44 (8.6)	5 (10.4)	0.5962
Age (years) (mean±SD)	36.66±12.13	36.32±12.03	40.45±12.81	0.8495
Multinodularity, n (%)	362 (64.29)	324 (63.0)	38 (79.2)	0.0250
Suppressed thyrotropin, n (%)	87 (15.4)	85 (16.5)	2 (4.2)	0.0241
Size of the largest nodule (cm), (mean±SD)	3.65±1.89	3.61 (1.87)	3.97 (2.08)	0.207
Suspicious cervical lymph node on USG, n (%)	4 (0.7)	1 (0.2)	3 (6.3)	-
Microcalcifications, n (%)	48 (8.5)	35 (6.8)	13 (27.1)	0.0003
Total thyroidectomy, n (%)	346 (61.4)	308 (59.9)	38 (79.2)	0.0086

**Table 2:** Binary logistic regression USG: Ultrasonogram p-value of < 0.05 is considered significant.

	Odds ratio (95% CI)	Adjusted odds ratio	p-value
Multinodularity	2.4687 (1.2016-5.0719)	2.1	0.022
Male	0.8785 (0.4689-3.3042)	-	-
Age: 21-59 years	0.9366 (0.4610-2.4726)	-	-
Thyrotoxicosis	0.2353 (0.0561-0.9870)	0.22	0.04
Nodule size > 4 cm (USG)	0.8171 (0.4514-1.4792)	-	-
Suspicious cervical lymph node (USG)	104.2584 (5.5238-1967.8026)	12.6	0.04
Microcalcifications	5.0833 (2.4663-10.4769)	3.7	<0.001
Total thyroidectomy	2.5333 (1.2008-5.3448)	-	-

## Discussion

We report a malignancy rate of 8.5% in Bethesda II nodules. The most common type of malignancy that was missed in FNAC but recognized in the final histopathology was PTC (87.5%). The presence of suspicious cervical lymph nodes, microcalcifications, and multinodularity were independent positive predictors of malignancy, whereas suppressed TSH was a negative predictor.

The prevalence of malignancy in our study was much higher than most often cited, 0-3%, risk of malignancy in Bethesda II thyroid nodules. Previous Indian studies have reported a prevalence of malignancy in 0-13% of patients with Bethesda II thyroid nodules, with a pooled prevalence of 3% (95% CI: 2%-4%) [11]. A higher prevalence in our study may be related to the inclusion of only those patients for whom histopathology reports were available. Several studies have reported a high prevalence of malignancy in Bethesda II nodules [12,13]. A previous Indian study, in which all thyroid nodules were subjected to histological examination, also reported a high rate of malignancy (12.9%) in Bethesda II nodules [14]. However, a few other similar Indian studies reported a low prevalence of malignancy (1.3%, 3.3%) in Bethesda II nodules [15,16]. The missed malignancy was most commonly PTC, which is the most common thyroid malignancy and is also well known for being a microcarcinoma that is more likely to be missed in FNA [8,17].

The reasons for missing malignancy in Bethesda II nodules may be multiple. In a study from Turkey, a lack of cytopathologists for analysis was the strongest risk factor for false-negative thyroid FNAC [18]. However, in our study, a cytopathologist reported all Bethesda II nodules, which negates such a role. A recent meta-analysis reported sampling error as the most common reason for false negativity [17]. It was also the most likely cause in our study. This sampling error occurred despite ultrasound-guided FNAC, which may suggest either erroneous sampling of adjacent normal tissue or that of only benign nodule[s] in patients with multiple nodules. In line with this possibility, multinodularity was a positive predictor of false negativity in our study.

The prevalence of thyroid cancer within a MNG has demonstrated a wide distribution of cancer rates, ranging from 3 to 35% [19]. In a recent meta-analysis, MNG was slightly more common than solitary thyroid nodules among patients evaluated for the risk of malignancy, as also noted in our study. However, the risk of malignancy was 20% lower in MNG in the meta-analysis [20]. This may indicate a more frequent environmental factor-related origin of MNGs that are more often noncancerous. In contrast, in our study, the risk of malignancy was higher in MNG. Unlike studies from Western countries, a study from Nigeria has shown a higher risk of malignancy in MNG than in solitary thyroid nodules, which may indicate a role for, yet unknown, environmental or ethnicity-specific genetic factors that may be associated with an increased risk of malignancy in MNG [20,21].

Notably, total thyroidectomy was a predictor of malignancy in the univariate analysis. Total thyroidectomy is more likely to reveal malignancy in unsuspected or unsampled nodules than hemithyroidectomy, which might have made it a positive predictor of malignancy in the univariate analysis. However, total thyroidectomy was not an independent predictor of malignancy in binary logistic regression. This may be due to the performance of total thyroidectomy mostly in patients with multinodularity, which might have attenuated the independent prediction of malignancy by the extent of surgery. A strong association between total thyroidectomy and multinodularity about malignancy risk was demonstrated in a recent study that reported malignancy in 57% of patients with MNG who underwent total thyroidectomy [22].

It is interesting to note that the strongest risk factor for malignancy in Bethesda II nodules was the presence of suspicious cervical lymph nodes. All three patients with false-negative FNAC but suspicious lymph nodes had multiple nodules, indicating multinodular disease as the most likely reason for false negative results. A suspicious cervical lymph node is a well-known risk factor for malignancy and is an indication for FNA irrespective of the thyroid nodule characteristics [23,24]. A negative FNA in such scenarios should warn regarding the possibility of missed malignancy, and consideration should be given to aggressive surgical management. Fortunately, the operating surgeon recognized the high risk of malignancy in these three patients and performed total thyroidectomy with neck node clearance. Another important sonographic feature that predicted malignancy in our patients was the presence of microcalcifications. Microcalcification is a consistent predictor of thyroid malignancy and is a part of TIRADS [8]. Among Bethesda III nodules, microcalcification is one of the strong predictors of malignancy and helps in decision-making for surgery [25]. Hence, the presence of microcalcification and suspicious cervical lymph nodes on neck ultrasound should prompt a potential sampling error even if FNAC is reported for Bethesda II.

Toxic thyroid nodules are associated with a 55% lower risk of malignancy than nontoxic nodules, and suppressed TSH with a hot nodule is not recommended for FNA [8,26]. We also found thyrotoxicosis to be a negative predictor of malignancy in our study. The size of thyroid nodules was a predictor of thyroid malignancy in several studies but with variable prediction [17,27,28]. However, the risk was higher in larger nodules in a systematic review [29]. In contrast, in our study, nodule size was not a significant factor for thyroid malignancy. Interestingly, a larger nodule size is associated with reduced accuracy of FNA, which often favors surgical intervention in FNA-benign/inconclusive/intermediate large nodules [17,29]. Younger patients and males have a higher risk of malignancy among thyroid nodules but were not associated with malignancy in our study [23,28,30].

A limitation of our study was that it is a retrospective study with inherent limitations. Second, the study was limited to patients with Bethesda category II nodules. Notably, details of TIRADS and/or sonological details, other than calcification, to determine TIRADS score (composition, echogenicity, shape, and margin) were available in only 27% of patients due to incomplete documentation of sonological findings in the medical records. However, from the year 2011, obtaining thyroid function tests and a thyroid ultrasound before FNAC is a standard protocol at the center, which indicates the availability of the above-mentioned sonological details in most patients to the pathologist performing and interpreting FNAC at the time of performing FNAC. Notably, FNAC was ultrasound-guided in 178 (49.2%) of MNG and 37 (18.4%) of solitary thyroid nodules. Ultrasound-guided FNAC is useful to select one or more suspicious nodules in patients with MNG to improve diagnostic accuracy. Ultrasound-unguided FNAC in only around half of the patients might have contributed to the high proportion of malignancy observed in Bethesda II nodules of this study. Third, the information on the family history of thyroid disease and radiation exposure was limited. Lastly, the proportion of males was low, which may be due to less frequent occurrence of thyroid nodules in men or greater healthcare seeking by women.

## Conclusions

We report a high prevalence of malignancy among South Indian patients with Bethesda II thyroid nodules. In patients with Bethesda II thyroid nodules, thyroid microcalcification, suspicious cervical lymph nodes on ultrasound, and multinodularity are associated with an increased risk of malignancy, whereas suppressed TSH is associated with a lower risk of malignancy. Further prospective studies are warranted to confirm the study observations.
